# Prehabilitation in high-risk patients scheduled for major abdominal cancer surgery: a feasibility study

**DOI:** 10.1186/s13741-022-00263-2

**Published:** 2022-08-23

**Authors:** Jamie L. Waterland, Hilmy Ismail, Catherine L. Granger, Cameron Patrick, Linda Denehy, Bernhard Riedel, on behalf of the Centre for Prehabilitation and Perioperative Care, Anna Beaumont, Emma Bruns, Kate Burbury, Danika Carty, Rani Chahal, Georgina Christelis, Sonia Coleman, Jessica Crowe, Lara Edbrooke, Melanie Fairweather, Maria Ftanou, Kate Graham, Travis Hall, Simon Harrison, Alexander Heriot, Yesim Karabiyik, Kay Kenchington, Amit Khot, Erika Kotowicz, Naomi Lawrance, Debra Leung, Iris Liu, Jenelle Loeliger, Fiona Lynch, Alicia Martin, Jamie Norman, Kat O’Brien, Tom Poulton, Christina Prickett, Ian Richardson, Catherine Sinton, Amanda Siu, Emily Traer, Anya Traill

**Affiliations:** 1 Peter MacCallum Cancer Centre, Department of Anaesthesia, Perioperative and Pain Medicine, Melbourne, Australia; 2 Department of Physiotherapy, The University of Melbourne, Melbourne, Australia; 3 The University of Melbourne, Centre for Integrated Critical Care, Melbourne, Australia; 4 Physiotherapy Department, The Royal Melbourne Hospital, Melbourne, Australia; 5 Peter MacCallum Cancer Centre, Division of Allied Health, Melbourne, Australia; 6 The University of Melbourne, Statistical Consulting Centre, School of Mathematics and Statistics, Melbourne, Australia; 7 The Sir Peter MacCallum Department of Oncology, University of Melbourne, Melbourne, Australia

**Keywords:** High-risk patients, Major surgery, Cardiopulmonary exercise testing, CPET, Prehabilitation

## Abstract

**Background:**

Patients presenting for major surgery with low cardiorespiratory fitness (deconditioning) and other modifiable risk factors are at increased risk of postoperative complications. This study investigated the feasibility of delivering prehabilitation in high-risk patients scheduled for major abdominal cancer surgery.

**Methods:**

Eligible patients in this single-center cohort study included patients with poor fitness (objectively assessed by cardiopulmonary exercise testing, CPET) scheduled for elective major abdominal cancer surgery. Patients were recruited to participate in a prehabilitation program that spanned up to 6 weeks pre-operatively and comprised aerobic and resistance exercise training, breathing exercise, and nutritional support. The primary outcome assessed pre-specified feasibility targets: recruitment >70%, retention >85%, and intervention adherence >70%. Secondary outcomes were assessed for improved pre-operative functional status and health-related quality of life and for postoperative complications.

**Results:**

Eighty-two (34%) out of 238 patients screened between April 2018 and December 2019 were eligible for recruitment. Fifty (61%) patients (52% males) with a median age of 71 (IQR, 63–77) years participated in the study. Baseline oxygen consumption the at anaerobic threshold and at peak exercise (mean±SD: 9.8±1.8 and 14.0±2.9 mL/kg/min, respectively) confirmed the deconditioned state of the study cohort. The retention rate within the prehabilitation program was 84%, with 42 participants returning for repeat CPET testing. While >60% of participants preferred to do home-based prehabilitation, adherence to the intervention was low—with only 12 (28%) and 15 (35%) of patients having self-reported compliance >70% with their exercise prescriptions.

**Conclusion:**

Our prehabilitation program in high-risk cancer surgery patients did not achieve pre-specified targets for recruitment, retention, and self-reported program adherence. These findings underpin the importance of implementation research and strategies for the prehabilitation programs in major surgery.

**Trial registration:**

Australian New Zealand Clinical Trials Registry (ACTRN12620000073909) retrospectively registered.

**Supplementary Information:**

The online version contains supplementary material available at 10.1186/s13741-022-00263-2.

## Introduction

Surgery remains the first line of treatment for two thirds of cancer types, and cancer surgery represents ~15% of all elective surgeries in Australia (Australian Institute of Health and Welfare 2016). Unfortunately, more than 25% of adult patients will suffer a major postoperative complication (e.g., myocardial injury, pulmonary complications, sepsis) within the first 30 days after surgery with far-reaching consequences (*Can Med Assoc J* 2019). For example, postoperative pulmonary complications, which occur in between 18 and 60% of patients following major abdominal surgery (Fernandez-Bustamante et al. 2017; Haines et al. 2013; Lockstone et al. 2020) associates with a 69% reduction in 30-day survival (Khuri et al. 2005) and prolonged ICU/hospital length of stay (Fernandez-Bustamante et al. 2017). Postoperative complications also significantly impact the healthcare system, with an estimated 20–30% of surgical costs attributed to postoperative complications (Weiser et al. 2015).

With an aging and increasingly sedentary population demographic, we can expect that a greater percentage of patients will present for surgery who are elderly, with increased comorbid disease burden including deconditioning and frailty and thus at increased risk of postoperative complications (Wynter-Blyth and Moorthy 2017; Older and Levett 2017). Most promising is that a number of recent studies report a substantial reduction in postoperative complications following targeted prehabilitation in patients with modifiable risk, including deconditioning (poor fitness levels), anemia, and malnutrition (Bolshinsky et al. 2018). Preoperative risk-stratification is therefore essential to identify high-risk patients, especially those who have a modifiable risk who can then be referred to prehabilitation programs (Levett et al. 2016; Glance et al. 2014).

Prehabilitation attempts to reverse modifiable risk within high-risk patients scheduled for major surgery. High-risk cohorts are identified using thresholds of age >70 and/or American Society of Anaesthesiologists (ASA) grading (Barberan-Garcia et al. 2018) or the presence of frailty (Carli et al. 2020). A more objective risk assessment though is afforded by cardiorespiratory exercise testing (CPET), with key variables (e.g., oxygen consumption at anaerobic threshold [AT] and at peak exercise [peak VO_2_]) reported to reliably predict postoperative morbidity and mortality (Older et al. 1999). Early studies by Older et al. (1999) and the more recent Measurement of Exercise Tolerance before Surgery (METS) study (Wijeysundera et al. 2018) report that an AT of <11mL/kg/min and a peak VO_2_ of <15mL/kg/min are predictive of postoperative mortality and non-cardiac complications, respectively. These thresholds have also been replicated in other surgical cohorts (Levett and Grocott 2015; Wilson et al. 2010), including major abdominal surgeries (Moran et al. 2016).

Prehabilitation is reported to minimize complications associated with low cardiorespiratory fitness (Carli and Scheede-Bergdahl 2015; Carli et al. 2017). Prehabilitation can include exercise prehabilitation (Boereboom et al. 2016), inspiratory muscle training (van Adrichem et al. 2014), preoperative respiratory education including breathing exercises (Boden et al. 2018), nutritional (Zhang et al. 2012), haematinic (Borstlap et al. 2015; Tonia et al. 2012), and psychological optimization (Tsimopoulou et al. 2015), and it is essential that prehabilitation programs move beyond unimodal programs to deliver multimodal bundles of care (Bolshinsky et al. 2018). Despite the emerging support for prehabilitation (The National Institute for Research, Royal College of Anaesthetists, and MacMillan Cancer Support 2019), key gaps in knowledge remain. Many current studies of prehabilitation contain small sample sizes (<40 participants) (Kim et al. 2009; Dunne et al. 2016; Cho 2004; West et al. 2014a) are non-randomized trials (Cho 2004; West et al. 2014a; Li et al. 2013), exclude patients with metastatic disease (Minnella et al. 2018), and/or include patients with higher baseline exercise capacity (Cho 2004). Systematic reviews reporting on the effects of prehabilitation suggest that patients with lower baseline fitness or physical function are likely to benefit most from prehabilitation programs (Bolshinsky et al. 2018; Minnella et al. 2016; Vermillion et al. 2018). That said, there is currently only one published protocol that aims to investigate prehabilitation in high-risk patients based on objective CPET markers with AT <11ml/kg (Berkel et al. 2018). Additionally, there is limited research that seeks to understand how prehabilitation may work in a real-world clinical setting (Older et al. 1999; Moran et al. 2016).

This study investigated the feasibility of delivering a hospital- and community-based prehabilitation program in patients identified at high risk of postoperative complications, based on objective baseline CPET testing, scheduled for major cancer surgery. Secondary aims explored the effects of prehabilitation on improving pre-operative cardiorespiratory fitness, physical function, health-related quality of life, exercise self-efficacy, and reducing postoperative complications.

## Methods

### Study design and setting

This prospective, single-arm, study investigated the feasibility of providing a prehabilitation program, with individualized exercise prescriptions, in patients scheduled for major abdominal cancer surgery who were identified as being at high risk for postoperative complications based on objective CPET-derived data informing of poor baseline functional capacity. The study was conducted at a tertiary/quaternary cancer center in Australia, with institutional ethics approval (LNR/18/PMCC/40).

#### Participants

Consecutive patients were screened for inclusion in this study at the pre-operative CPET Clinic. Patients being considered for major abdominal cancer surgery are routinely referred to the CPET clinic for risk stratification, to assess their suitability for surgery and for postoperative destination planning (e.g., surgical ward, extended recovery unit, HDU, or ICU). Patients were eligible for inclusion if they satisfied the inclusion and exclusion criteria presented in Table 1. Inclusion criteria were intentionally broad to include a real-world sample of high-risk patients preparing for major cancer surgery. Following agreement to participate, all patients were required to provide written informed consent. Participants then attended an appointment with a physiotherapist for baseline assessment of functional capacity (including a 6-min walk test (6MWT), handgrip strength testing, and 30-s sit to stand test), their individualized exercise prescription, respiratory exercises, and malnutrition screening. Participants identified as at risk of malnutrition on screening and/or in need of psychology input were referred to the dietetics and psychology services as part of standard care pathways.

**Table 1:** Inclusion and exclusion criteria

Inclusion criteria	
• Planned to have major intra-abdominal cancer surgery, defined as >2-h duration and requiring an overnight hospital stay • Aged ≥ 18 years • English speaking • >2 weeks prior to surgery • Outpatients or in-patients with planned discharged from the hospital in less than 1 week • Cardiopulmonary exercise testing results of AT ≤11ml/kg/min and/or VO2 peak ≤15ml/kg/min and/or VO2 peak ≤710ml/min/m2. Patients that were unable to complete CPET were included if they had ≤70% predicted distance on the 6MWT	
Exclusion criteria	
• Myocardial infarction in the last 3 months • Unstable angina • Cerebrovascular event or transient ischemic attack in the last 3 months • Pulmonary embolic event within 3 months • Existing acute or chronic deep vein thrombosis • Pregnancy • Presentation with active sepsis • Planned for surgery in < 2 weeks • Hospital inpatient, with anticipated admission >1 week • Unable or contra-indication to exercise • Unable to exercise unsupervised without appropriate supervision for safety reasons	

### Prehabilitation program

#### Exercise intervention

**Fig. 1:**
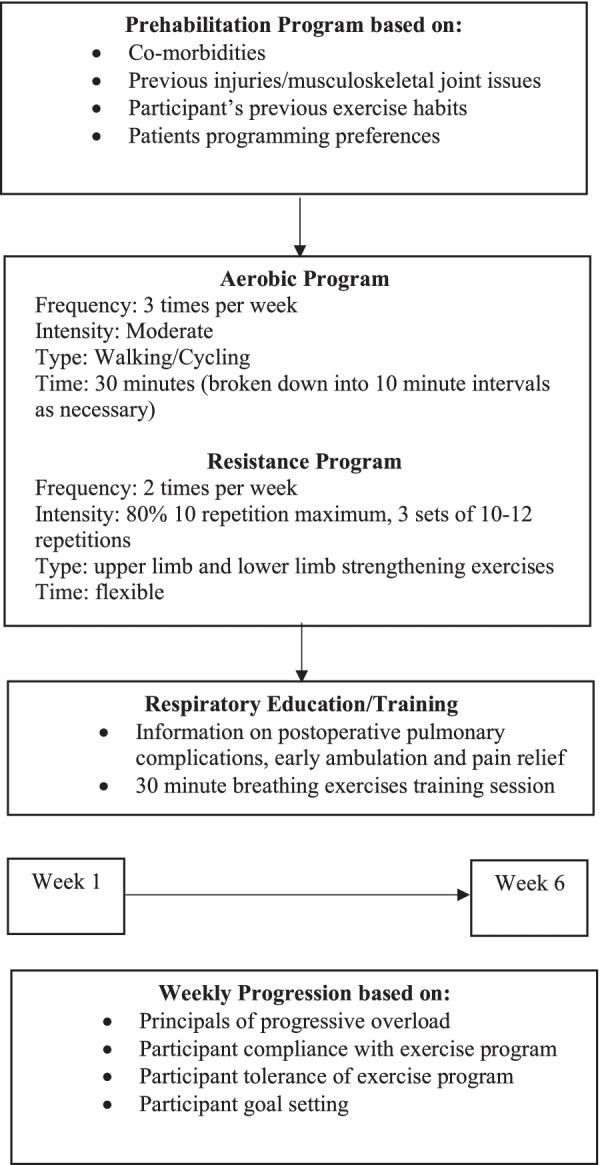
Prehabilitation program flowchart

Participants were prescribed an individualized exercise program based on the American College of Sports Medicine (ACSM) exercise guidelines for patients with cancer (Schmitz and Speck 2010) (Fig. 1). Given the complexity of this study population, exercise prescription was individualised to each person, with particular adaptions based on previous musculoskeletal injuries/issues (e.g., total joint replacements), symptoms such as pain and the patient’s exercise preferences. Exercise programs were prescribed and then progressed by a qualified physiotherapist or exercise physiologist based on participants’ response to exercise and adherence to the current exercise program. Goal setting and continued rating of self-efficacy allowed for individualized exercise interventions to be progressed and modified to achieve participant recognized important goals. Participants were given the choice of completing their exercise programs at the hospital or in settings closer to or within their home (e.g., home-based or community gyms). Further detail regarding the exercise intervention is provided as per the TIDieR checklist (Hoffmann et al. 2014) in Supplement 1.

#### Breathing exercise intervention

Information on postoperative pulmonary complications and the importance of early postoperative ambulation, good pain relief, and breathing exercise prescriptions to prevent these complications were provided to all patients. Breathing exercises were based on the Active Cycle of Breathing (ACBT) technique (Lewis et al. 2012) and were provided in an educational session with practice supervised by a senior physiotherapist or senior nurse with experience in perioperative care.

#### Usual care

Patients who were not eligible for recruitment into this study received standard/usual preoperative care. Standard care prior to March 2019 included a recommendation for pre-operative exercise advice as part of the pre-admission clinic, but this did not include supervised exercise training or physiotherapy sessions in the weeks leading up to surgery. Patients non-agreeable to the study after March 2019 were offered the same program of preoperative physiotherapy including preoperative exercise training, information/education, and screening for preoperative nutritional needs as the study participants through standard care as part of a newly established multidisciplinary prehabilitation service.

#### Postoperative care

Postoperatively, study participants received standard postoperative care that included routine postoperative physiotherapy on postoperative day one and then ongoing physiotherapy as needed for respiratory management and early mobilization and individualized to patient needs. Following discharge from the hospital, standard care did not include referral to a standardized postoperative exercise program and is representative of current standard practice in Australia.

### Study outcome measurements

Primary study outcomes assessed for study feasibility, and secondary outcomes assessed for improved functional outcomes, patient well-being, and for incidence of postoperative complications. Functional outcomes were measured at baseline and the following prehabilitation. Postoperative complications were assessed at 30 days after surgery. Outcome assessors were not blinded to participation in the research study.

#### Feasibility (primary outcome)

Feasibility was assessed by recruitment (number of patients screened and/or consented) and retention (number of participants who attended repeat CPET assessments), adherence with the intervention (based on compliance with the exercise prescription and attendance at breathing education sessions). To be considered feasible to design a larger more definitive efficacy study, we set the target for recruitment >70% of eligible participants consenting to enrol in the prehabilitation program (Thabane et al. 2010). Additionally, retention was assessed with a target of >85% of participants attending a second CPET test; and adherence with the prehabilitation program was assessed against the target of individuals achieving >70% of the prescribed exercise program (self-reported) during the study follow-up with phone calls.

#### Cardiopulmonary fitness

CPET was performed at baseline and at the end of the prehabilitation program as per the Perioperative Exercise Testing and Training Society (POETTS) practice guidelines (Levett et al. 2018). Tests were analyzed by anaesthetists accredited in CPET assessment. Gas exchange-derived variables were obtained during a ramp protocol with a cycle ergometer to ascertain participants’ cardiorespiratory fitness. Traditional CPET-derived parameters that were analyzed included oxygen consumption (VO_2_) at anaerobic threshold (AT; ml/kg/min) and at peak exercise (pVO_2_) corrected to both patient body weight (ml/kg/min) and to patient body surface area (ml/min/m^2^). VO_2_ at AT was determined according to the POETTS guidelines, using the three-point estimate of modified V-slope, ventilatory equivalents, and increasing end-tidal partial pressure of oxygen (P_ET_O_2_) (Levett et al. 2018). The risk of postoperative pulmonary complications was assessed using the Assess Respiratory Risk in Surgical Patients in Catalonia (ARISCAT) Score (Canet et al. 2010).

#### Functional exercise capacity and strength

Functional exercise capacity was assessed objectively using the 6-min walk test (6MWT) according to the American Thoracic Society guidelines (American Thoracic Society 2002). Physical Activity Levels were assessed subjectively by the International Physical Activity Questionnaire (IPAQ) (Craig et al. 2003). Handgrip strength was assessed on both dominant and non-dominant hands using a hydraulic handheld dynamometer (Baseline® Lite™ Hydraulic Hand Dynanometer, Baseline® Evaluation Instruments, Fabrication Enterprises, White Plains, NY10602, USA). Participants were given three attempts with both hands and the maximum reading recorded. Lower body strength was assessed using a sit-to-stand test over 30 seconds (Rikli and Jones 1999). Participants were instructed to sit in a standardized chair and stand up and sit down as many times as possible within 30 seconds.

#### Health-related quality of life (HRQoL) assessment

HRQoL was measured by the Functional Assessment of Cancer Therapy – General (FACT-G) (Cella et al. 2002). The total scores vary from 0 to 136, with higher scores indicating a better the quality of life. The Edmonton Symptom Assessment Scale (ESAS) was used to describe symptoms commonly seen in cancer patients. It involves eight visual analog scales (VAS) to indicate levels of pain, activity, nausea, depression, anxiety, drowsiness, appetite, and sensation of wellbeing (Bruera et al. 1991). A self-efficacy questionnaire was also administered (Rogers et al. 2006). This survey includes a nine-item scale that measures the most common barriers to exercise reported among cancer patients (including lack of discipline, nausea, exercise not a priority, bad weather, fatigue, lack of interest, time, lack of enjoyment, and lack of encouragement) and a four-item task self-efficacy which asked participants to rate their confidence in the ability to walk 20 min without stopping, run for 10 min without stopping, climb three flights of stairs without stopping, and exercise for 20 min at a level hard enough to cause a large increase in heart rate and breathing (Rogers et al. 2006). Self-efficacy was rated on a scale from 0 to 100% at 10% intervals. General headings were also provided as guides (not at all confident, 0–20%: slightly confident, 20–40%; moderately confident, 40–60%; very confident, 60–80%; extremely confident, 80–100%). The Malnutrition Screening Tool (MST), a simple, quick, valid, and reliable tool, was used to identify patients at risk of malnutrition (score of ≥2) (Ferguson et al. 1999).

#### Postoperative outcomes

Postoperative outcomes that were measured included ICU admission and length of stay, days until the participant sat out of bed, medical emergency team (MET) calls during hospital admission, hospital length of stay, hospital re-admissions, and a patient centric measure—days at home within 30 days after surgery (DAH-30) (Myles et al. 2017). Postoperative pulmonary complications were assessed using the Melbourne Group Score (MGS version 2) (Parry et al. 2014) and postoperative complications were graded by the Clavien-Dindo scoring system (Dindo et al. 2004).

### Statistical analysis of secondary outcomes

Given that this was a feasibility study, we did not undertake formal a priori power calculation. Rather, we recruited a study size of convenience that reflected our target population of high-risk patients deemed candidates for prehabilitation (Thabane et al. 2010). This feasibility study was not powered to test efficacy, did not include a control group, and we did not adjust for multiple factors because the aims of the study were exploratory. Continuous variables are reported as mean (range), mean (standard deviation, SD) or median (interquartile range, IQR), depending on distribution, and categorical variables as frequency (number, %). Change over time in physical activity, functional exercise capacity, strength, mood, and HRQoL of participants exposed to the intervention were examined from the baseline to completion of the intervention assessment time point (prior to surgery) and analyzed using paired samples *t* test. Linear mixed models were used to investigate change over time across the three timepoints; baseline, end of intervention (preoperative, prior to surgery), and 30 days postoperatively for 6MWT and handgrip strength testing. Regression analyses initially planned were not conducted as there was not enough data to make these analyses meaningful. All analyses were performed with the statistical software IBM SPSS Statistics Version 25 (SPSS© IBM Corp). Participants still awaiting surgery were followed up for a period of 100 days after the completion of the prehabilitation intervention prior to study closure. Participants who did not have their surgery by this follow up timepoint were not included in the postoperative analysis (*n* = 2 patients).

## Results

Of the 50 participants who consented to participate in the study, 26 (52%) were male and their median age was 71 (IQR: 63–77) years. Further demographic data are reported in Table 2. The majority of participants had colorectal cancer (*n*=32, 64%). Given the study eligibility criteria, all participants were deconditioned, as confirmed by baseline average oxygen consumption at AT of 9.8 (SD, 1.8) and average oxygen consumption at peak exercise (peak VO_2_) of 14.0 (SD, 2.9) mL/kg/min. Forty participants (80%) reported they were not currently exercising. All patients were either at intermediate (*n*=27, 54%) or high (*n*=23, 46%) risk for postoperative pulmonary complications based on their ARISCAT risk scores. Sixteen participants (40%) were at risk of malnutrition based on the MST.

**Table 2:** Participant characteristics

	Overall (*n*=50)
Gender (male, *n* (%))	26 (52%)
Age (years), median [IQR]	71 [63–77]
Age (years), range	44–84
Colinet Comorbidity Score, median [IQR]	10.5 [6.75–12.25]
ARISCAT, *n* (%)	
Intermediate risk	27 (54%)
High risk	23 (46%)
At risk of malnutrition, *n* (%)^a^	
Yes	16 (40%)
No	27 (63%)
Cancer type, *n* (%)	
Colorectal	32 (64%)
Oesophageal	9 (18%)
Sarcoma	6 (12%)
Pancreatic	1 (2%)
Prostate	1 (2%)
Gastric	1 (2%)
Surgery type, *n* (%)	
Bowel resection	12 (24%)
Oesophagectomy	9 (18%)
Cytoreductive surgery with HIPEC	8 (16%)
Pelvic exenteration	6 (12%)
Ultra-low anterior resection	6 (12%)
Sarcoma resection	3 (6%)
Pancreatectomy	1 (2%)
Prostatectomy	1 (2%)
Gastrectomy	1 (2%)
Debulking surgery	1 (2%)
Reversal of Hartmans	1 (2%)
Whipples operation	1 (2%)
CPET-derived variables	
AT (mL/kg/min), mean±SD	9.8±1.8
VO_2_ Peak (mL/kg/min), mean±SD	14.0±2.9
VO_2_ Peak/BSA (mLs/kg), mean±SD	578±121
Smoking status, *n* (%)	
Never smoked	13 (26%)
Quit smoking >2 months	32 (64%)
Current smoker	5 (10%)
Living situation, *n* (%)	
Living alone	9 (18%)
Living with family	41 (82%)
Residing >30km from hospital, *n* (%)	34 (68%)
Reported currently not exercising, *n* (%)	40 (80%)

*Abbreviations*: *ARISCAT* Assess Respiratory Risk in Surgical Patients in Catalonia, *BSA* body surface area, *CPET* cardiopulmonary exercise test, *HIPEC* hyperthermic intraperitoneal chemotherapy. ^a^*n*=43, 7 missing Malnutrition Screening Tool Results

### Feasibility

**Fig. 2:**
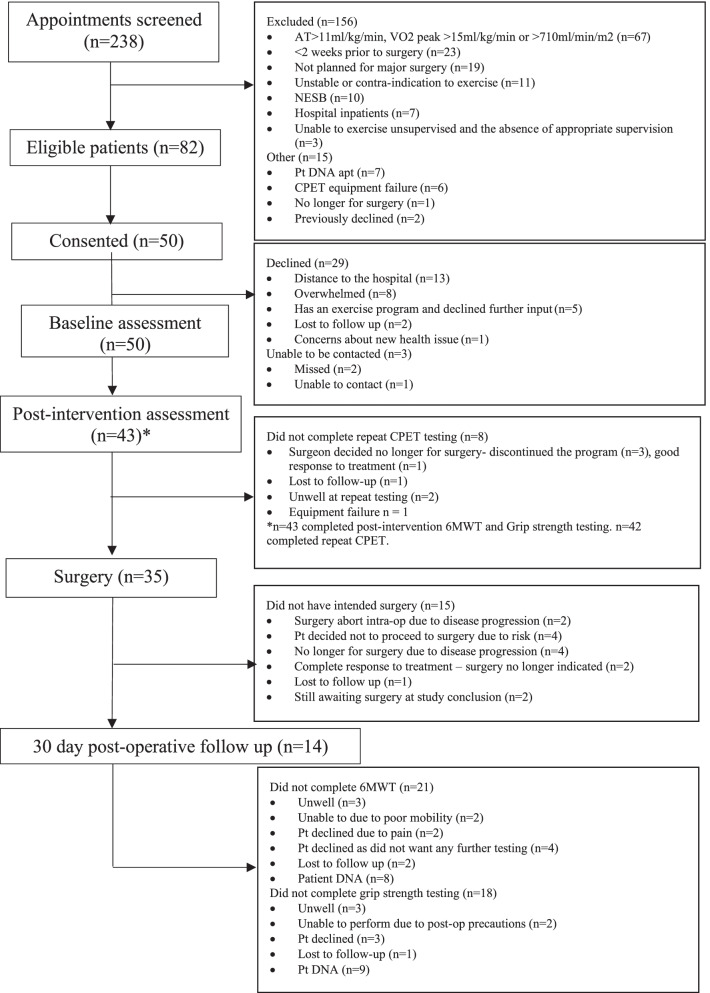
Participant flowchart. Abbreviations: CPET cardiopulmonary exercise test, DNA did not attend, NESB non-English speaking background, Pt patient, VO_2_ volume of oxygen, 6MWT 6-min walk test

Two hundred and thirty-eight potential participants were screened between April 2018 and December 2019, of these 82 (35%) were deemed eligible for recruitment into the study and 50 participants (61%) consented to participate in the study (Fig. 2). Twenty-nine (35%) potential participants declined to participate, and three patients could not be contacted. The main reasons for the decline were distance to the hospital (*n*=13, 45%) and feeling overwhelmed (*n*=8, 28%), while some patients already had an exercise program and did not want additional input (*n*=5, 17%).

Of the 50 participants who consented to participate, 44 (88%) patients attended their exercise prescription appointment, 36 (72%) attended their respiratory exercise education session, 43 (86%) completed repeat functional assessments (6MWT and handgrip strength), 42 (84%) had repeat CPET testing, and 35 (70%) proceeded to their planned surgery.

Approximately one-quarter of the study participants had altered indications for surgery: disease progression (*n*=6; 40%), change of plan due to the risk of surgery (*n*=4; 27%), no longer indicated due to response to treatment (*n*=2; 13%). At study’s conclusion two patients were still awaiting surgery and one participant was lost to follow-up.

Of the 44 participants who attended their exercise prescription session, 27 (61%) of participants chose to complete their exercise sessions within their own home (Table 3). Barriers to completing the exercise program reported by patients were fatigue, conflicting appointments or traveling for appointments, busy with other commitments (e.g., family illnesses), illness (e.g., common cold), symptoms of cancer (e.g., pain), weather, boredom, and poor motivation when set-backs occurred (e.g., surgery date postponed). There was one adverse event that occurred during the exercise intervention, one participant experienced hypotension following the exercise session that was related to underlying medical disease and required overnight admission.

**Table 3:** Program compliance and adherence

	*n* (%)
Received respiratory intervention	36 (72%)
Attended exercise prescription appointment	44 (88%)
Primary exercise program location preference	
Home-based	27 (61%)
Community gym	5 (11%)
Hospital gym	9 (21%)
Community centre/local hospital	2 (5%)
Other (private PT)	1 (2%)
Duration of prehabilitation program (mean, weeks)^a^	5.9
>70% compliance with 3x week aerobic exercise	12 (28%)
>70% compliance with 2x week strength exercise	15 (35%)

^a^In the 43 patients that completed their program including repeat functional exercise testing

### Exploratory findings

The primary aim of this study was to assess feasibility and not intervention efficacy; hence, this study was underpowered to detect statistically significant differences in exploratory outcomes and instead we report on trends. Mean scores for objectively measured fitness and functional capacity are presented in Table 4.

**Table 4:** Functional assessment at baseline and post-exercise intervention

Outcome measures	Baseline	Post-intervention	Mean difference	95% Confidence interval
CPET				
AT (ml/kg/min)	9.7 (1.6)	10.6 (1.8)	0.9	0.3, 1.5
Peak VO_2_ (ml/kg/min)	13.8 (3.0)	14.7 (3.0)	0.8	0.2, 1.4
Peak VO_2_/BSA (mLs/kg)	581 (127.5)	625 (143.6)	43.6	11.6, 75.6
Sit-to-stand (repetitions)	12 (4)	12 (4)	0	1, 2
	*n*	Mean		95% Confidence interval
6MWD (m)				
Baseline	43	451		418.6, 482.3
Post-intervention	43	471		437.8, 503.3
30 days postoperative	28	349		308.1, 390.7
Handgrip strength (kg)				
Baseline	43	34		30.3, 36.9
Post-intervention	43	35		31.4, 38.1
30 days postoperative	29	31		27.0, 34.8

Data are presented as mean (standard deviation). Paired sample *t* test used for CPET variables (*n*=42) and sit-to-stand (*n*=27) variables and linear mixed models used for 6MWD (*n*=43) and handgrip strength (*n*=43). Abbreviations: *6MWD* 6-min walk distance, *CPET* cardiopulmonary exercise test, *AT* anaerobic threshold, *VO*_*2*_ *peak* volume of O_2_ consumed at peak exercise, *BSA* body surface area

Change in functional capacity, as assessed by follow-up CPET assessment, demonstrated a trend for improvement in AT and peak VO_2_ between baseline and end of the prehabilitation intervention phase, with a mean improvement of 0.9 (95%CI 0.32–1.51) and 0.8 (95%CI 0.17–1.39) mL/kg/min, respectively. However, this did not reach the minimally clinically important difference (MCID) reported in other prehabilitation studies of 1.5–2.0 mL/kg/min (Dunne et al. 2016; West et al. 2014b).

Mixed models, with time as a fixed effect and participant as a random effect, were used to explore change in 6MWD and handgrip strength over time. The observed mean difference (95% CI) between baseline and end of intervention with prehabilitation for 6MWD was 20 (95% CI −3.26–43.38) m, with large inter-patient variability. The best handgrip strength, irrespective of the hand, between baseline and end of intervention showed little change, with a mean difference of 1.1 (95% CI −0.98–3.18) kg.

No change was found in health-related quality of life score (Table 5). However, there was a trend for a reduction in severity of common symptoms on the Edmonton Symptom Assessment Scale (ESAS). Participants also indicated on International Physical Activity Questionnaire (IPAQ) of an increase in vigorous activity per week after completion of the program. There were no changes in self-reported self-efficacy

**Table 5:** Health-related quality of life at baseline and post-exercise intervention

Median (SD)	Baseline	Post-intervention	95% Confidence Interval
FACT-G questionnaire total score (*n*= 25)	79 (15)	85 (15)	−4.1, 7.2
Physical well-being	22 (5)	24 (3)	−4.1, 0.1
Social and family well-being	23 (5)	23 (6)	−2.5, 3.0
Emotional well-being	17 (5)	19 (4)	−4.2, 0.8
Functional well-being	17 (7)	20 (6)	−6.1, 0.8
ESAS total (*n*=25)	22 (11)	21 (11)	−11.8, −0.5
IPAQ (*n*=29)			
Total MET minutes/week	2305 (2878)	3858 (4236)	−2718, −388
Vigorous MET minutes/week	961 (1730)	1902 (2410)	−1598, −283
Moderate MET minutes/week	466 (582)	931 (1233)	−928, −2.6
Walking MET minutes/week	877 (1132)	1023 (1193)	−642, 349
Sitting hours/day (*n*=22)	5.3 (2.4)	5.4 (3.4)	−1.3, 1.2
Barrier to self-efficacy (*n*=25)			
Overall score	442 (244)	550 (299)	−267.3, 51.4
Task self-efficacy (*n*=21)			
Overall score	302 (349)	212 (121)	−85.7, 265.6

*Abbreviations: FACT-G* The Functional Assessment of Cancer Therapy – General, *ESAS* Edmonton Symptom Assessment Scale, *IPAQ* International Physical Activity Questionnaire, *MET* metabolic equivalent

### Post-operative outcomes

Thirty-five participants (70%) proceeded to the initially intended surgery during the study period (Fig. 2). Participant median [IQR] length of hospital stay was 14 [7, 25] days. Eighteen (53%) were admitted to ICU, and the median length of ICU stay was two days. All participants were on average out of bed the day after surgery, and three patients were re-admitted to hospital within 30 days post discharge. Participants days at home (DAH-30: median (range)) was 10 (0–26) days within the 30-day follow-up time period.

More than two thirds (*n*=22, 69%) of patients suffered at least one postoperative complication. The most common complications were gastrointestinal and hematological complications followed by cardiovascular postoperative complications, and only one (3%) participant experienced a postoperative pulmonary complication as per the Melbourne Group Score (Table 6). Complications ranged in severity; however, Grade II Clavien Dindo classification, requiring pharmacological treatment with drugs other than such allowed for grade I complications, was the most common (48%).

**Table 6:** Postoperative outcomes (*n*=35)

	*n* (%)
Length of hospital stay, median [IQR]	14 [7,25]
Admission to ICU, *n* (%)	18 (52%)
Length of ICU stay, median [IQR]	2 [1,2]
MET calls during admission, *n* (%)	4 (11%)
Days until SOOB, median [IQR]	1 [1,2]
DAH-30, days [IQR]	10 [2, 21]
DAH-30, days, Range	0-26
Readmission, *n* (%)	3 (9%)
PPC, *n* (%)	1 (3%)
Type of complication, *n* (%)^a^	
Gastrointestinal	9 (41%)
Hematological	9 (41%)
Cardiovascular	5 (23%)
Pulmonary	4 (18%)
Renal	4 (18%)
Infectious	3 (14%)
Wound	3 (14%)
Pain	3 (14%)
Highest grade of Clavien-Dindo complication severity by participant, *n* (%)	
Grade I	2 (10%)
Grade II	10 (48%)
Grade IIIa	4 (19%)
Grade IIIb	3 (14%)
Grade IVa	2 (10%)

*Abbreviations*: *DAH-30* days at home within 30 days after surgery, *PPC* postoperative pulmonary complications using the Melbourne Group Score (Parry et al. 2014). ^a^Percentages add up to >100% as patients two most severe complications were recorded

## Discussion

Despite finding trends of improvement in AT and peak VO_2_ on CPET, increased self-reported physical activity and a reduction in symptom severity following prehabilitation in this cohort of high-risk patients prior to major cancer surgery recruitment fell just short of our pre-selected target of >70% thought to be needed to design a larger more definitive trial of this prehabilitation intervention (Thabane et al. 2010).

Our recruitment rate of 61% is similar to that observed by other cancer surgery groups (Brahmbhatt et al. 2020) and significantly higher than 18%, 35%, and 49% reported in other studies involving preoperative exercise interventions prior to elective colorectal surgery (Northgraves et al. 2019; Karlsson et al. 2019) and in patients with advanced cancer (Sheill et al. 2019), respectively. Our original aim of >70% may have been too ambitious in this study population of elderly high-risk patient population, with the majority doing no exercise at all. It is also unclear whether the establishment of our prehabilitation clinic as standard care during the study period improved or hindered recruitment.

Of the 50 participants that consented to participate, the majority attended the exercise prescription appointment (88%) and received the respiratory exercises (72%), and 84% attended the second CPET clinic within 5.9 weeks on average following their first CPET appointment. The attrition rate of three-quarters of the study population (74%) to attend the postoperative timepoint was very high. The reasons for drop out to the last study timepoint were participants were medically unwell, had limited mobility, pain, and not wanting any further testing or did not attend the scheduled postoperative appointment. These have previously been cited in the literature as barriers to participation in clinical trials, especially exercise trials (Sheill et al. 2019; Ormel et al. 2018). The high attrition to the post-operative timepoint was similar to that reported in advanced cancer patients (Sheill et al. 2019) and may be reflective of the substantial burden of major surgery, the need for adjuvant treatment, and/or additional surgery or experiencing prolonged recoveries.

Overall self-reported adherence to the intervention in this study was half of that expected, with more participants reporting adherence to resistance than to aerobic exercises. Reasons for this included personal factors such as fatigue and physical limitations due to comorbid health conditions are consistent with other studies within older patients awaiting abdominal surgery (Agasi-Idenburg et al. 2020). There is little research examining patient preference between anaerobic and resistance training programs whilst experiencing symptoms of cancer and/or during cancer treatment (Courneya et al. 2008); however, older adults with colorectal cancer when interviewed advocated for exercise programs that could be provided close to home. Thus, it may not be surprising that participants may be more adherent to resistance exercise that can be done within the home, without the need to leave the home to access specialised equipment (e.g., exercise bikes) or contend with the weather. A recent study that interviewed patients with cancer awaiting major abdominal surgery also noted that being at home felt safe, especially when suffering with physical symptoms such as nausea or diarrhea or psychological issues such as anxiety (Beck et al. 2020) and this may be an additional reason for this result.

Additional barriers specific to the preoperative colorectal cancer surgery population include the acknowledgement of the need for an exercise program. A study by Agasi-Idenburg et al. (2020) noted that older patients awaiting colorectal cancer surgery often use their normal daily activities as a proof of adequate level of physical fitness and may not acknowledge the need for an exercise program, even if recommended by a health care professional, if they consider themselves sufficiently active. A qualitative study of 16 patients awaiting major abdominal surgery (Beck et al. 2020) demonstrated that prehabilitation programs were only part of preparing for surgery and that patients had very clear opinions about what was important to them in the preoperative period, and this included prioritizing time with loved ones, preparing meals, and the house for the postoperative period and preparing a will. This prioritization of competing tasks may have contributed to the adherence results found in our study. Perhaps more education about the importance of exercise in aiding recovery may have improved this result. The importance of preoperative education was highlighted in a recent study where postoperative pulmonary complications were reduced by 50% following one physiotherapy education session (Boden et al. 2018). Similarly, a study of 144 patients awaiting major abdominal surgery in which the prehabilitation program included motivational interviewing to assess adherence profile and codesign characteristics of the physical activity program with the patient found a significant reduction in postoperative complications (31% versus 62%) (Barberan-Garcia et al. 2018).

Incorporating exercise education or “Surgery School” into routine cancer care (Grocott et al. 2017), engaging family support (Ormel et al. 2018), regularly reinforcing the importance of exercise at preoperative “teachable moments” as well as at each preoperative clinical contact with the range of healthcare professionals in the surgical multidisciplinary team (medical, nursing, and allied health staff) may support patients towards positive behaviour change earlier in the preoperative pathway (Grocott et al. 2017; Robinson et al. 2020). The inclusion of follow-up reminders with the use of digital technologies may even further promote change (Robinson et al. 2020).

The majority of participants chose to exercise within the home-based environment (61%), with 68% of our study participants residing >30km away from the treating hospital, and thus adherence to exercise program intensity was unable to be supervised and accurately monitored within this study and data are subject to recall bias. This is consistent with contemporary published literature (Agasi-Idenburg et al. 2020; Beck et al. 2020; Waterland et al. 2020). Previous studies suggest that the intensity of exercise required to facilitate cardiorespiratory exercise benefits may be unable to be achieved within the home-based setting without supervision (Edbrooke et al. 2019). Therefore, it may be essential to adapt existing interventions to facilitate improved exercise fidelity and patient adherence to exercise programs within the major surgery group such as the inclusion of a technology-driven exercise interventions to more accurately monitor exercise program adherence, such as those described by Steffens et al. (2020).

Of the enrolled patients, 16% did not proceed to planned surgery due to tumour progression or increased medical risk. This underpins the importance of ensuring prehabilitation programs do not delay surgery but rather are co-designed with surgeons to ensure patients are referred for prehabilitation at the earliest timepoint possible, even before diagnostic workup and neoadjuvant therapy is commenced (if indicated). That is, modifying comorbid disease with prehabilitation programs should be executed in parallel to the diagnostic workup of the surgical disease to avoid any delay. As a consequence, some patients who will commence a prehabilitation program may be deemed not suitable/not requiring surgery. Importantly, if prehabilitation reduce postoperative complications and expedite postoperative recovery, this may also improve access times to postoperative adjuvant therapy and facilitate better cancer outcomes.

### Strengths and limitations of the study

This study had several strengths including investigating the use of a prehabilitation intervention in a high-risk population within a real-world clinical context and the inclusion of a number of exploratory outcomes, which provide pilot data for sample-size calculations for future studies. The lack of a control group makes it impossible to comment on intervention efficacy, but trends in improvement across patient trajectories warrant further investigation. Further strategies to improve recruitment and adherence within this group are warranted within this high-risk group and warrant studies of implementation and psychological motivation in prehabilitation. Other limitations include the lack of objective exercise training data and the reliance of self-reporting of exercise adherence and physical activity levels which, while common in exercise oncology literature, are often misreported (Nicolson et al. 2018). Additionally, the role of neoadjuvant treatment in the preoperative period was not quantified. It may be possible that the absence of a decline in AT and peak VO_2_ during this period may have demonstrated the beneficial effect of prehabilitation in those patients undergoing neoadjuvant therapies, which can result in loss of functional capacity by as much as 20% (West et al. 2014a).

## Conclusion

Our data suggest that a prehabilitation program and recruitment strategies used within this study for high-risk patients preparing for major abdominal cancer surgery did not achieve our pre-specified targets (70%) for recruitment and adherence. For patients preparing for major cancer surgery, prehabilitation may facilitate improvements in cardiorespiratory fitness and functional ability in the preoperative period. While these findings are encouraging, and largely reflect previous prehabilitation research, they point to a need for refining processes and resources when planning implementation and clinical trials on prehabilitation to suite the requirements of local conditions and patient populations. Also, adequately powered trials of prehabilitation interventions of high-risk patients that are deconditioned at baseline are needed to confidently determine intervention effectiveness in the preoperative time period available.

## Supplementary Information

**Additional file 1.** TIDieR checklist.

## Data Availability

Data supporting the conclusions of this article are available from the PI on request.
