# Supplementary material for: Prehabilitation in high-risk patients scheduled for major abdominal cancer surgery: a feasibility study
Source: Perioper Med (Lond). 2022 Aug 23;11:32. doi: 10.1186/s13741-022-00263-2 (PMC9396890; doi:10.1186/s13741-022-00263-2)
Supplement: Supplementary file 1 — Additional file 1. TIDieR checklist. [file 13741_2022_263_MOESM1_ESM.docx]

**TIDieR checklist: Multimodal Prehabilitation in High Risk Cancer Surgery Intervention**

| **1** | *Brief Name*  *Provide the name or a phrase that describes the intervention.* | Multimodal Prehabilitation in High Risk Cancer Surgery |
| --- | --- | --- |
| **2** | *Why*  *Describe any rationale, theory, or goal of the elements essential to the intervention.* | Refer to manuscript background |
| **3** | *What – Materials*  *Describe any physical or informational materials used in the intervention, including those provided to participants or used in intervention delivery or in training of intervention providers. Provide information on where the materials can be accessed (e.g. online appendix, URL).* | Participants were provided with an individualised home-based exercise prescription on paper by the physiotherapist or exercise physiologist prescribing the exercises.  Participants were also provided with a handout of the breathing exercises recommended as part of the intervention (provided as part of Supplement 1). |
| **4** | *What – Procedures*  *Describe each of the procedures, activities, and/or processes used in the intervention, including any enabling or support activities.* | Whole body exercise intervention including aerobic and resistance exercises.  Pre-operative education including breathing exercises, education regarding early post-operative mobility and pain relief. |
| **5** | *Who Provided*  *For each category of intervention provider (e.g. psychologist, nursing assistant), describe their expertise, background and any specific training given.* | Senior Physiotherapist, tertiary qualified exercise professional with clinical experience in exercise prescription of cancer patients prior to surgery.  Senior Exercise Physiologist, tertiary qualified exercise professional with clinical experience in exercise prescription of cancer patents prior to surgery.  Exercise Programs were prescribed and progressed by either a Senior Physiotherapist or Senior Exercise Physiologist based on staff availability and was not captured as part of the study analysis.  Senior Nurse, tertiary qualified health professional with clinical experience in preoperative oncology nursing. |
| **6** | *How*  *Describe the modes of delivery (e.g. face-to-face or by some other mechanism, such as internet or telephone) of the intervention and whether it was provided individually or in a group.* | Face-face exercise prescription appointment  Choice of face-face, community based gym/community centre or home based unsupervised exercise sessions  Telephone follow up phone calls for those completing home based exercise sessions. |
| **7** | *Where*  *Describe the type(s) of location(s) where the intervention occurred, including any necessary infrastructure or relevant features.* | Tertiary hospital gym  Community health centre or gym  Home based sessions |
| **8** | *When and How Much*  *Describe the number of times the intervention was delivered and over what period of time including the number of sessions, their schedule, and their duration, intensity or dose.* | Aerobic and Resistance Exercise Program delivered over 6 weeks  3 x per week, 1hr sessions  Intensity: moderate  One-off 30min pre-operative education session including information regarding breathing exercises, early post-operative ambulation and pain relief. |
| **9** | *Tailoring*  *If the intervention was planned to be personalised, titrated or adapted, then describe what, why, when, and how.* | Exercise intervention was individualised based on co-morbidities, previous injuries/musculoskeletal joint issues, previous exercise habits and patient’s exercise programming preferences.  The same preoperative education was provided to all participants.  Aerobic exercise was prescribed to moderate intensity of walking/cycling for a duration of 30 mins based on RPE.  Resistance exercises were prescribed at 80% of 10 repetition maximum for 3 sets of 10-12 reps for major UL/LL muscle groups. |
| **10** | *Modifications*  *If the intervention was modified during the course of the study, describe the changes (what, why, when, and how).* | Exercise intervention was progressed by the study physiotherapist or exercise physiologist to ensure sessions were conducted at a moderate intensity.  Aerobic exercise: distance/speed was increased as participants tolerated sessions.  Resistance exercise: weight was increased when 3 sets 10-12 reps could be completed. |
| **11** | *How Well - Planned*  *If intervention adherence or fidelity was assessed, describe how and by whom, and if any strategies were used to maintain or improve fidelity, describe them.* | Adherence was assessed by participant self-report upon weekly phone call for those participants completing their progress at home and in person at exercise sessions for those attending the hospital.  Goal setting and continued rating of self-efficacy was used to support participant adherence to the exercise intervention. |
| **12** | *How Well – Actual*  *If intervention adherence or fidelity was assessed, describe the extent to which the intervention was delivered as planned.* | Please see results section of manuscript |
